# Supplementary material for: Nucleocapsids of the Rift Valley fever virus ambisense S segment contain an exposed RNA element in the center that overlaps with the intergenic region
Source: Nat Commun. 2024 Sep 1;15:7602. doi: 10.1038/s41467-024-52058-2 (PMC11365940; doi:10.1038/s41467-024-52058-2)
Supplement: Supplementary file 1 — Supplementary Information [file 41467_2024_52058_MOESM1_ESM.pdf]

Nucleocapsids of the Rift Valley fever virus ambisense S segment contain an exposed RNA element in the center that overlaps with the intergenic region

Lyudmila Shalamova[1], Patrick Barth[2,8], Matthew J. Pickin[1], Kiriaki Kouti[1], Benjamin Ott[3], Katharina Humpert[3,9], Stefan Janssen[4], Gema Lorenzo[5], Alejandro Brun[5], Alexander Goesmann[2], Torsten Hain[3], Roland K. Hartmann[6], Oliver Rossbach[7], and Friedemann Weber[1*]

[1]Institute for Virology, FB10-Veterinary Medicine, Justus-Liebig University, D-35392 Giessen, Germany.

[2]Bioinformatics & Systems Biology, Justus-Liebig University, D-35392 Giessen, Germany.

[3]Institute for Medical Microbiology, FB11-Medicine, Justus-Liebig University, D-35392 Giessen, Germany.

[4]Algorithmic Bioinformatics, Justus-Liebig University, D-35392 Giessen, Germany.

[5]Centro de Investigación en Sanidad Animal (CISA-INIA/CSIC), Valdeolmos 28130 Madrid, Spain.

[6]Institute of Pharmaceutical Chemistry, Philipps-University Marburg, Marbacher Weg 6, D-35037 Marburg, Germany.

[7]Institute for Biochemistry, FB 08-Biology and Chemistry, Justus-Liebig University, D-35392 Giessen, Germany.

[8]Current address: Cell Biology and Plant Biochemistry, University of Regensburg, Universitätsstrasse 31, 93053 Regensburg, Germany

[9]Current address: Institute of Molecular Oncology, Genomics Core Facility, Philipps-University, 35043 Marburg, Germany

*corresponding author: Tel.: +49-641 9938350; Fax: +49-641 9938359

E-mail: friedemann.weber@vetmed.uni-giessen.de

Supplementary figures

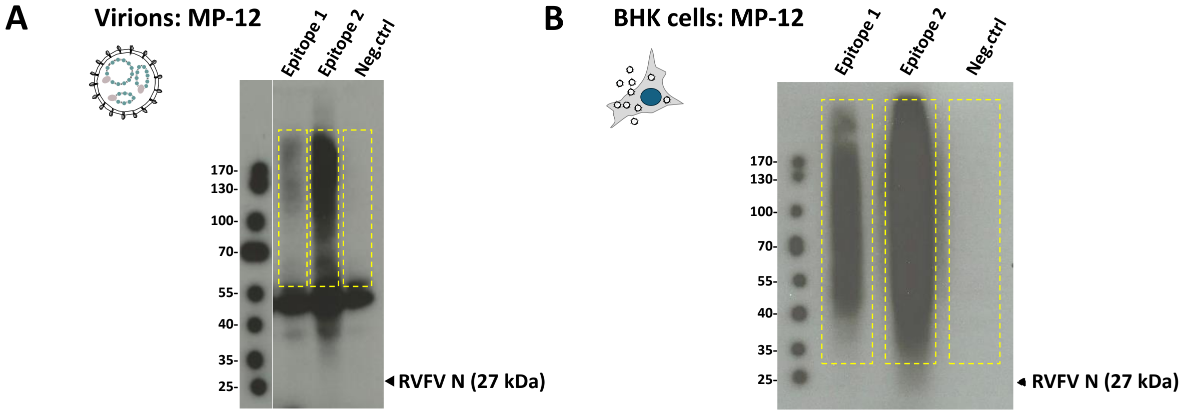

Figure S1: RVFV nucleoprotein iCLIP.

Autoradiography of ^{32}P-labelled RNA covalently bound to enriched RVFV N following immunoprecipitation using distinct monoclonal antibodies against RVFV N ("Epitope 1", "Epitope 2") or negative control antibody ("Neg.ctrl"). Representative images for (A) RVFV MP-12 virions, (B) MP-12 infected BHK cells (5 h p.i) are shown. Masks for material excision from the membrane following RNA transfer are depicted by yellow dashed boxes. All experiments were performed in three biological replicates. Source data are provided as a Source Data file.

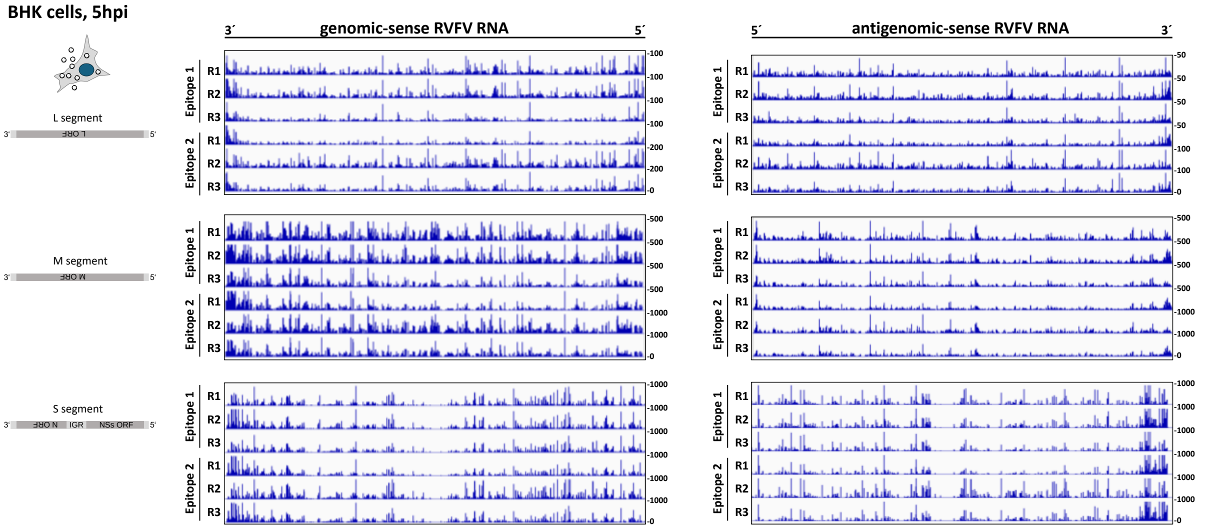

Figure S2: Mapping of RVFV nucleoprotein-RNA interaction by iCLIP in early infection. IGV tracks representing read numbers of crosslink sites (Y-axes) at single nucleotide resolution along the RNA segment sequences of RVFV MP-12 (X-axes), obtained by iCLIP in infected BHK cells (5 h p.i). For each of the viral segments (L, M and S, as schematically indicated on the left) viral RNA in genomic-sense orientation is shown 3'-5' and 5'-3' in antigenomic-sense orientation. Two distinct monoclonal antibodies were used for iCLIP experiments and data from three biological replicates ("R1-R3") are shown for each of them ("Epitope 1", "Epitope 2"). Track height range (crosslink site count) is adjusted by autoscale and presented on the right side of each track. For further information, see legend to Fig. 2 of the main text.

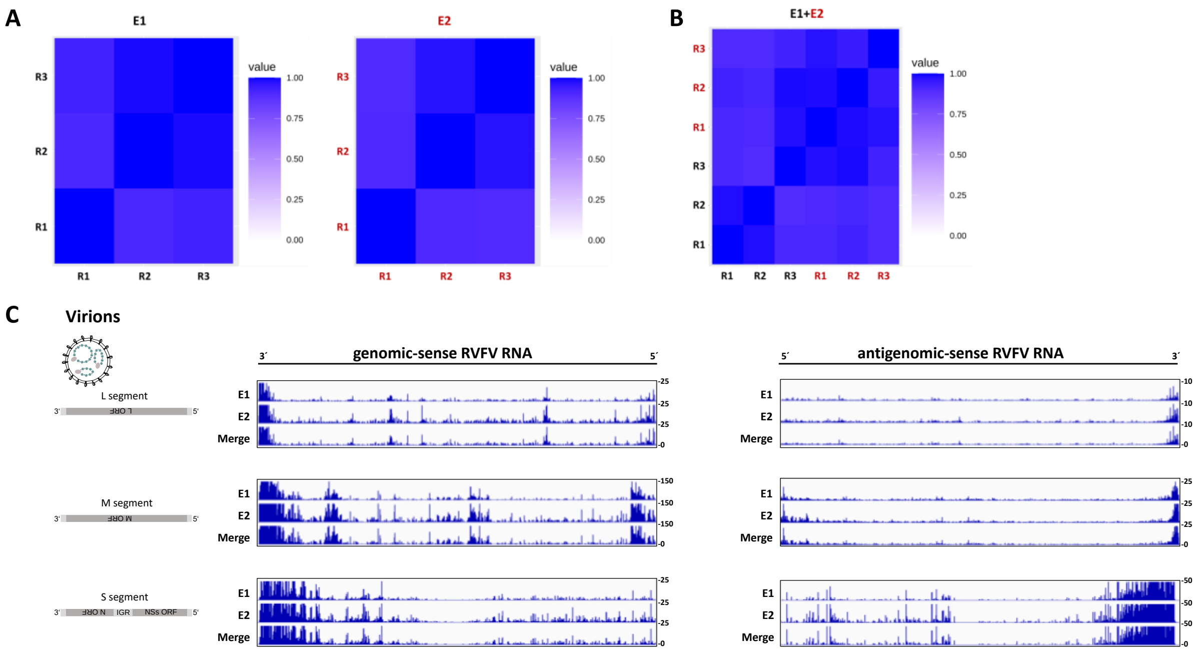

Figure S3: RVFV nucleoprotein-RNA interaction in virions.

Heatmaps illustrate (A) the similarity between three biological replicates obtained for RVFV nucleoprotein iCLIP in MP-12 virions, for each of the two monoclonal antibodies used ("E1", "E2"), and (B) the similarity between the data for the two epitopes compared to each other ("E1+E2"). (C) IGV tracks represent an average of the three biological replicates for each epitope ("E1, E2") and an average of all six biological replicates for both epitopes ("Merge"). The tracks are grouped by RVFV segment (L, M and S) and viral RNA in genomic orientation is shown 3'-5' and in antigenomic - 5'-3'. Track height range (crosslink site count) is shown on the right side of each track.

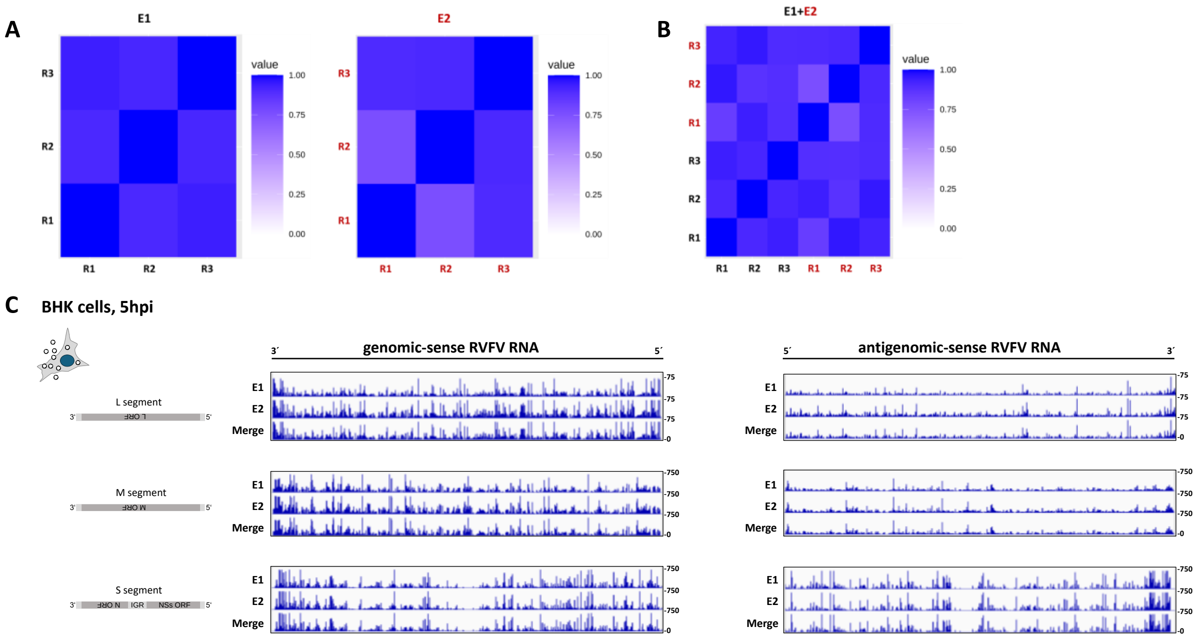

Figure S4: RVFV nucleoprotein-RNA interaction in infected BHK cells.

Heatmaps (A, B) and IGV tracks (C) for the three biological replicates obtained for RVFV nucleoprotein iCLIP in BHK cells infected with MP-12 (5 h p.i), with the same setup as for virion iCLIP (see Fig. S3).

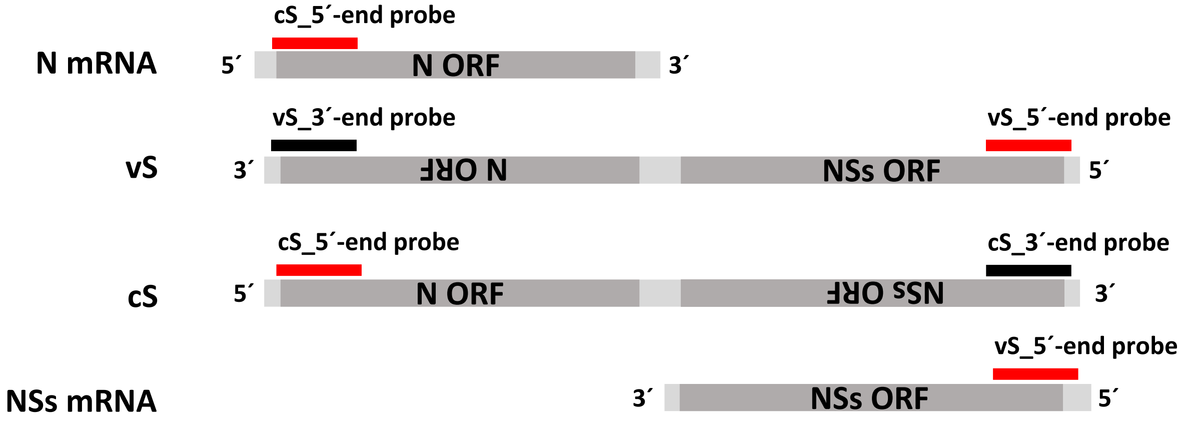

Figure S5: Positions of probes used to detect S segment genomic and antigenomic RNA by northern blotting.

Schematic representation of the RVFV S segment RNA species: genomic ("vS") and antigenomic ("cS") RNA, as well as the corresponding mRNAs. For each RNA, coding (in dark grey) and untranslated (in light grey) regions are schematically depicted as well as binding positions for the vS/cS 3' end probes (thick black lines) and for the vS/cS 5' end probes (thick red lines). Note that only the vS/cS 5' end probes also recognise the mRNAs.

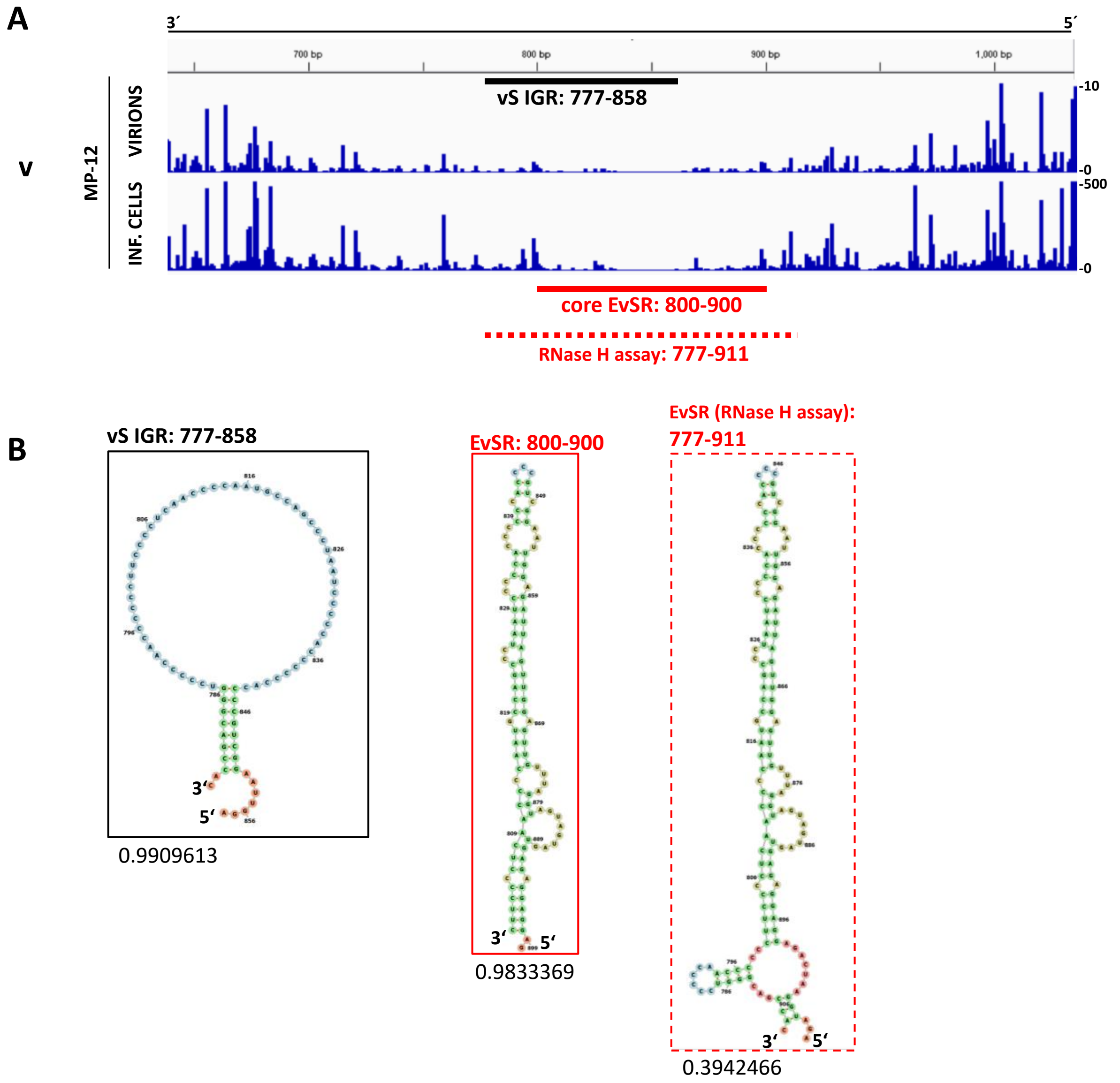

Figure S6: RNA secondary structure prediction for RVFV RNA exposed in vS segment vRNP.

(A) IGV tracks summarize N binding profiles within a fragment of MP-12 viral genomic RNA (~650-1050) obtained for virions and in infected BHK cells. Regions corresponding to the IGR (black line), the EvSR (solid red line) and the exposed region identified by the RNase H protection assay (dashed red line) are shown. (B) RNA minimal free energy secondary structure prediction for the three highlighted RNA sequences within RVFV genomic RNA as described in (A). The structures with the highest probability (indicated below the structures) are shown for each RNA sequence.

A

EvSR: 800-900

```
800         3’ CUUCCCCUCAACCCCAAUGCCAGCCCUAAUCCCCCACCCCCCACCCCGUCGGAAUUGGAGAUUAGUUGGAGUUGUUUAGGUAGUAGUAGUGAGAGGAGGAG 5’      900
-26.00         (((((.(((((((.((((.(((((..(((((..(((...((.((...)).))...))).)))))))))).))))....)))........)))).)))))..         0.9833369 []
-22.50         (((((.((((.((.((((.(((((..(((((..(((...((.((...)).))...))).)))))))))).))))....))((....)).)))).)))))..         0.0166631 [[][]]
```

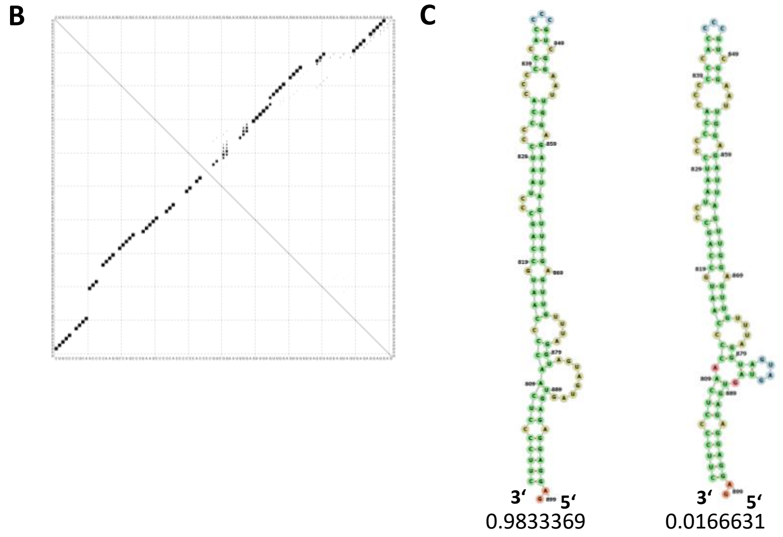

Figure S7: RNA secondary structure ensemble of the core EvSR.

(A) RNA structure probabilities (right column) for the core EvSR of RVFV MP-12, as computed by “RNAshapes” in probabilities mode via BiBiServ (see Materials and Methods). The minimal free Gibb‘s energy (left column) secondary structure for each shape class is reported as representative in Dot-Bracket notation. (B) Base pair probabilities of structure ensemble visualized as Dot Plot (upper right triangle) and maximum expected accuracy secondary structure (lower left triangle), which incorporates maximal compatible base pair probabilities. (C) “Forna” visualisations of shape representatives from (A), together with their accumulated shape class probability. Sequence for the genomic-sense RNA is shown 3’-5’ and nucleotide positions are presented as in the reference genome.

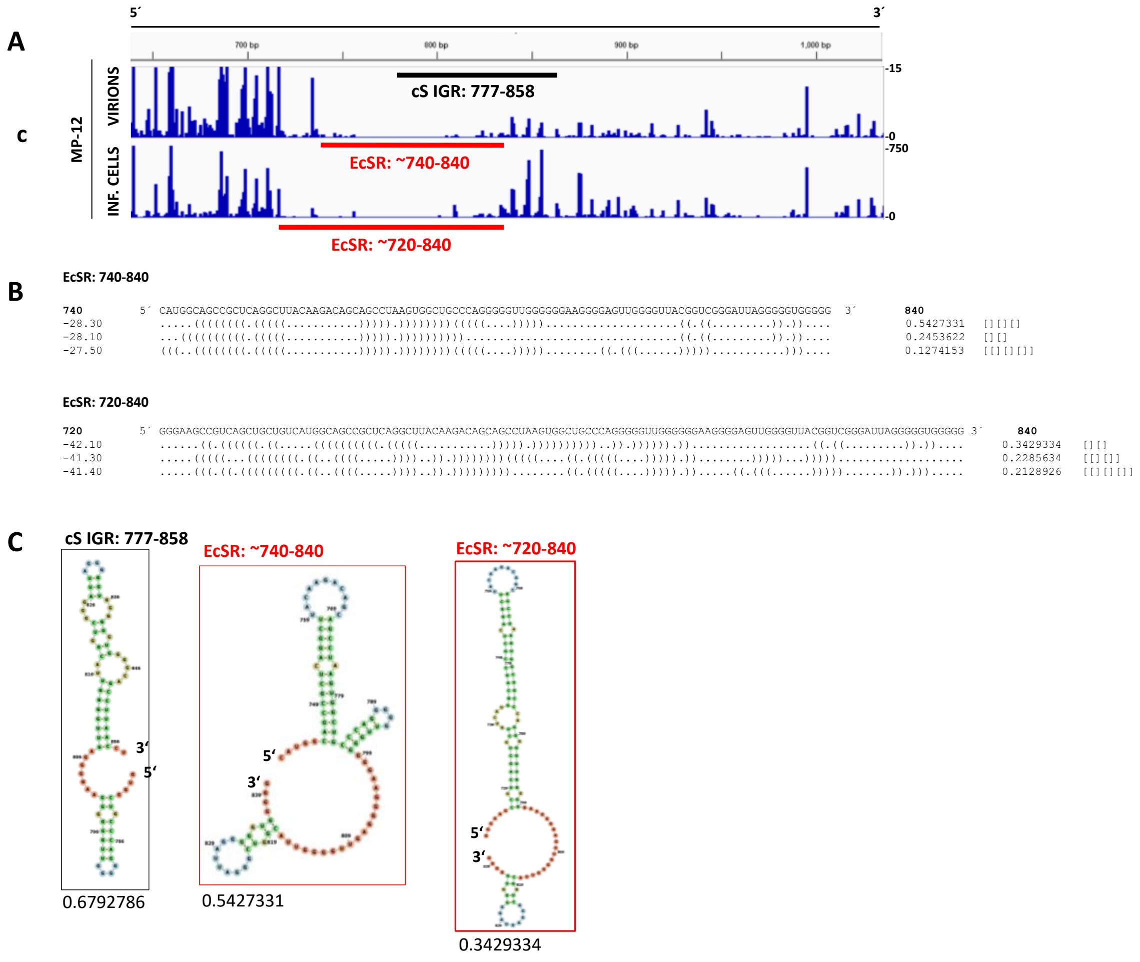

Figure S8: RNA secondary structure prediction for RVFV RNA exposed in cS segment vRNP.

(A) IGV tracks summarizing the iCLIP-derived N binding profiles within a fragment of MP-12 viral antigenomic RNA obtained for both virions and infected BHK cells. Regions corresponding to the antigenomic intergenic region (IGR, positions 777-858; black line) and to the proposed exposed cS regions (EcSR; solid red line) are shown individually for virions and infected cells system (positions ~740-840 and ~720-840, respectively). (B) RNA shape class probabilities (right values) for the EcSR of MP-12 (top – predicted for virions and bottom – for infected cells) were calculated using the probability mode of the BiBiServ "RNAshapes" studio with default parameters. Secondary structures are representatives of classes with lowest free Gibb's energy (left values) per class. List is reduced to top 3 classes, which constitutes more than 70% total probability mass. (C) RNA minimal free energy secondary structure prediction

by RNAfold (visualized via „forna“) for the RNA sequences within RVFV antigenomic RNA as highlighted in (A). The structures with the highest probability (indicated below the structures) are shown for each RNA sequence. Sequence for the antigenomic-sense RNA is shown 5’-3’ and nucleotide positions are presented as in the reference genome.

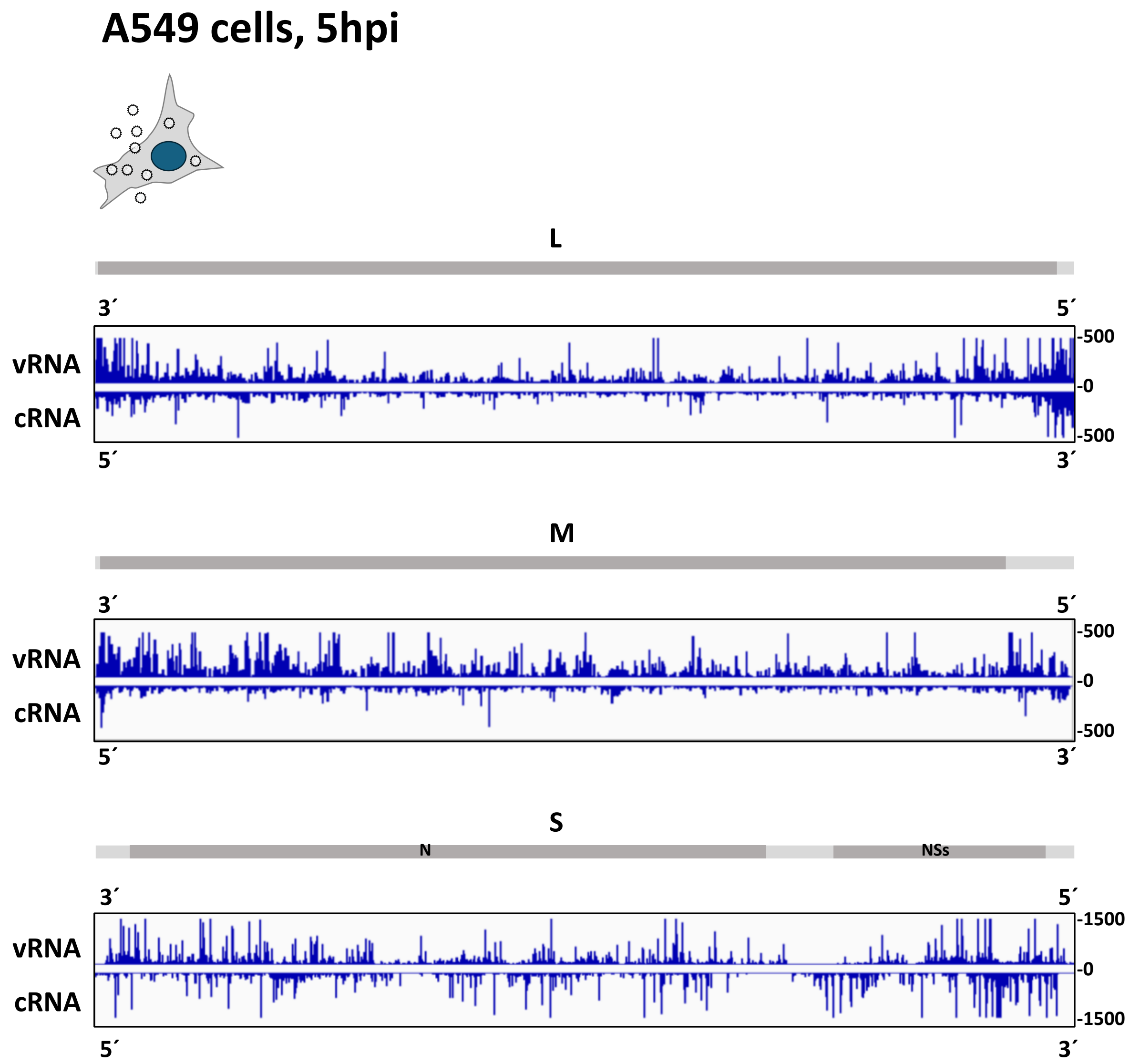

Figure S9: Nucleoprotein-RNA interaction in RVFV Clone 13-infected A549 cells.

IGV tracks summarizing RVFV N binding along all vRNA and cRNA segments in Clone 13-infected A549 cells (5 h p.i). Tracks are derived as average of three biological replicates and grouped by RVFV segment (L, M and S), illustrated as in the preceding iCLIP figures.

EvSR

777 … 910

3′CACCGACGGGUCCCCAACCCCCCUUCCCCUCAACCCCAAUGCCAGCCCUAAuCCCCCACCCCCCACCCCGUCGGAAUUGGAGAUUAGUUGGAGUUGUUUAGGUAGUAGUAGUGAGAGGAGGAGACUAAGGUAGA 5′

N side mutant:

3′CACCGACGGGUCCCCAACCCCCuccuuCucugguuCuggcGuugauCCcggcuCCuugCCCuuCguCCCGUCGGAAUUGGAGAUUAGUUGGAGUUGUUUAGGUAGUAGUAGUGAGAGGAGGAGACUAAGGUAGA 5′

NSs side mutant:

3′CACCGACGGGUCCCCAACCCCCCUUCCCCUCAACCCCAAUGCCAGCCCUAAuCCCCCACCCCCCACCCCacCaaAAUcaaAagUcgaccaaAaccaUUUAaacAGUAGUAGcagaAaagaaAGACUAAGGUAGA 5′

N/NSs side mutant:

3′CACCGACGGGUCCCCAACCCCCuccuuCucugguuCuggcGuugauCCcggcuCCuugCCCuuCguCCCacCaaAAUcaaAagUcgaccaaAaccaUUUAaacAGUAGUAGcagaAaagaaAGACUAAGGUAGA 5′

Nucleotides predicted to be base paired
NSs "ORF"
mutated nucleotides

Figure S10: Mutations introduced into the EvSR.

Sequence of the wt EvSR (top) and the mutant sequences employed for the experiments shown in main figure 9.

Supplementary tables

Table S1. RVFV MP-12 Virion N-iCLIP2 adapter barcodes [20] at the 5' end in sequencing and input barcodes

Replicate	Sample	Barcode name	Sequence (5' to 3')
1	Antibody 1	L15	NNNNNTGACATNNNN
1	Antibody 2	L10	NNNNNAAGCTANNNN
2	Antibody 1	L23	NNNNNATCGTGNNNN
2	Antibody 2	L03	NNNNNGCCTAANNNN
3	Antibody 1	L02	NNNNNACATCGNNNN
3	Antibody 2	L08	NNNNNTCAAGTNNNN
1	Input	NEBNext Index 30	NNNCCGGTGNNN
2	Input	NEBNext Index 38	NNNAGCTAGNNN
3	Input	NEBNext Index 48	NNNTCGGCANNN

Table S2. RVFV MP-12-infected cells N-iCLIP2 adapter barcodes [20] at the 5' end in sequencing

Replicate	Sample	Barcode name	Sequence (5' to 3')
1	Antibody 1	L01	NNNNNCGTGATNNNN
1	Antibody 2	L02	NNNNNACATCGNNNN
2	Antibody 1	L04	NNNNNTGGTCANNNN
2	Antibody 2	L08	NNNNNTCAAGTNNNN
3	Antibody 1	L10	NNNNNAAGCTANNNN
3	Antibody 2	L15	NNNNNTGACATNNNN
1	Size-matched input	L17	NNNNNTAGTTGNNNN
2	Size-matched input	L21	NNNNNCGAAACNNNN
3	Size-matched input	L23	NNNNNATCGTGNNNN

Table S3. RVFV Clone 13-infected A549 cells N-iCLIP2 adapter barcodes [20] at the 5' end in sequencing

Replicate	Sample	Barcode name	Sequence (5' to 3')
1	Antibody 3	L02	NNNNNACATCGNNNN
2	Antibody 3	L01	NNNNNCGTGATNNNN
3	Antibody 3	L16	NNNNNGGACGGNNNN
1	Size-matched input	L15	NNNNNTGACATNNNN
2	Size-matched input	L02	NNNNNACATCGNNNN
3	Size-matched input	L01	NNNNNCGTGATNNNN

Table S4: DNA oligonucleotides for northern probe template generation (synthesized by Microsynth)

Oligo ID	Sequence (5’ to 3’)	Purpose
vS_NB_5’end_for	GGGCCCTGTTGTGTCTTTCT	PCR template generation for vS 5’ end probe
vS_NB_5’end_rev_SP6	ATTTAGGTGACACTATAGAAA GCCAGTGAGGGTTCTCC	
vS_NB_3’end_for	GTTGATGAGAGCCTCCACAGTT	PCR template generation for vS 3’ end probe
vS_NB_3’end_rev_SP6	ATTTAGGTGACACTATAGAAC AGTGGGTCCGAGAGTTTG	
cS_NB_5’end_for	GGGTTTGATGCCCGTAGAGT	PCR template generation for cS 5’ end probe
cS_NB_5’end_rev_SP6	ATTTAGGTGACACTATAGGCCA CTCACTCAAGACGACC	
cS_NB_3’end_for	AAAGCCAGTGAGGGTTCTCC	PCR template generation for cS 3’ end probe
cS_NB_3’end_rev_SP6	ATTTAGGTGACACTATAGGGG CCCTGTTGTGTCTTTCT	
vM_NB_for	AACTCTCTGCCAAGCCCATC	PCR template generation for vM probe
vM_NB_rev_T7	TAATACGACTCACTATAGGGC AGGCCATGAGCACACACTA	

Table S5. DNA antisense oligonucleotides (ASOs) (synthesized by Microsynth)

ASO ID	Sequence (5’ to 3’)	Target in RVFV MP-12 vS (DQ380154.1) 5’ to 3’ (3’ to 5’)
Scrambled	TTCGTCTTGATAAAGCTCCCC	no target
#1	ATAAGAACAATGAGGGCTGA	671-690 (1001-1020)
#2	GGATTGGAGGAATAACTGGA	751-770 (921-940)
#3	CTCCTCCTCTGATTCCATCT	780-799 (892-911)
#4	GGGGTGGGGCAGCCTTAACCT	833-853 (838-858)
#5	TTGGGGTTACGGTCGGGATTA	862-882 (809-829)
#6	GTGGCTGCCCAGGGGGTTGGG	894-914 (777-797)
#7	AGGCTTACAAGACAGCAGCC	918-937 (754-773)
#8	AGCCATGAGAAGAGGAGAGA	1006-1025 (666-685)
